# Tracheitis Caused by Coinfection with Cytomegalovirus and Herpes Simplex Virus

**DOI:** 10.3390/medicina57111162

**Published:** 2021-10-26

**Authors:** Yu-Mi Lee, So-Woon Kim, Won-Gun Kwack

**Affiliations:** 1 Division of Infectious Diseases, Department of Internal Medicine, Kyung Hee University Hospital, Kyung Hee University School of Medicine, Seoul 02447, Korea; cristal156@hanmail.net; 2 Department of Pathology, Kyung Hee University Hospital, Kyung Hee University School of Medicine, Seoul 02447, Korea; sowoonkim86@gmail.com; 3 Division of Pulmonary, Allergy and Critical Care Medicine, Department of Internal Medicine, Kyung Hee University Hospital, Seoul 02447, Korea

**Keywords:** cytomegalovirus, herpes simplex virus, tracheitis

## Abstract

Clinically significant isolated viral tracheitis is scarce in adults, and upper airway obstruction caused by viral tracheitis is even more infrequent. A 74-year-old woman, who was administered low-dose steroids for two months for chronic obstructive pulmonary disease (COPD), developed dyspnea with stridor and required mechanical ventilation for respiratory failure. Chest computed tomography showed a diffuse tracheal wall thickening with luminal narrowing and peribronchial consolidation in the right upper lobe. Bronchoscopy revealed a proximal tracheal narrowing with multiple ulcerations of the tracheal mucosa surrounded by an erythematous margin. Pathologic examinations of the tracheal mucosal tissue, including immunohistochemistry, revealed a cytomegalovirus (CMV) and herpes simplex virus (HSV) infection. Furthermore, the bronchial alveolar lavage fluid was positive on the CMV real-time polymerase chain reaction. The patient was treated with intravenous ganciclovir for 44 days. The follow-up bronchoscopy 49 days after the initiation of ganciclovir revealed improved multiple ulcerations with scars. We report a rare case of tracheitis caused by coinfection with CMV and HSV in a patient with COPD who had been taking low-dose steroids for months. The case showed that CMV and HSV are potential causes of serious tracheitis and respiratory failure.

## 1. Introduction

Infectious tracheitis does not commonly develop in isolation, as it usually occurs with the inflammation of nearby organs, so-called laryngotracheitis or tracheobronchitis [1]. It can be caused by bacteria, viruses, and fungi [1,2]. Infectious tracheitis often improves spontaneously, but it can lead to life-threatening upper airway obstruction and post-infectious tracheal stenosis [1,2]. The exact incidence of infectious tracheitis in adults is unknown, because the symptom is usually mild in adults and the diagnosis is difficult without an invasive procedure such as bronchoscopy [1,2]. In terms of bacterial tracheitis, *Staphylococcus aureus*, *Haemophilus influenza*, and Group A *Streptococcus* are common pathogens, typically affecting children aged 6–10 years [1,3]. It is uncertain whether bacterial tracheitis is a primary or secondary infection that develops following mucosal damage due to viral infection. Otherwise, acute viral laryngotracheitis, commonly known as “croup,” causes stridor and is more common in children aged below six years [4]. Respiratory viruses, such as parainfluenza virus 1 and 2, influenza virus, respiratory syncytial virus, adenovirus, rhinovirus, coronavirus, and enterovirus, are the common viral pathogens of tracheitis, accompanied by laryngitis or bronchitis in children [5]. The risk of severe symptoms of upper airway infection in children is explained by the anatomic differences between children and adults, where the narrowing portion of the airway caused by inflammation occurs in the subglottis and glottis, respectively [1]. The subglottic region is composed of a loose mucous membrane that is prone to swelling when it is infected. The low incidence and the minor symptoms of viral tracheitis in adults may also be attributed to the body’s immunological memory [5]. Clinically significant isolated viral tracheitis is uncommon in adults, and upper airway obstruction caused by viral tracheitis is even more infrequent. We present a case of tracheitis caused by coinfection with cytomegalovirus (CMV) and herpes simplex virus (HSV), which resulted in respiratory failure. This study was approved by the Institutional Review Board (KHUH-2021-07-047) of Kyung Hee University Hospital, Seoul, Korea, which waived the need for written informed consent from the patients.

## 2. Case Report

**Figure 1:**
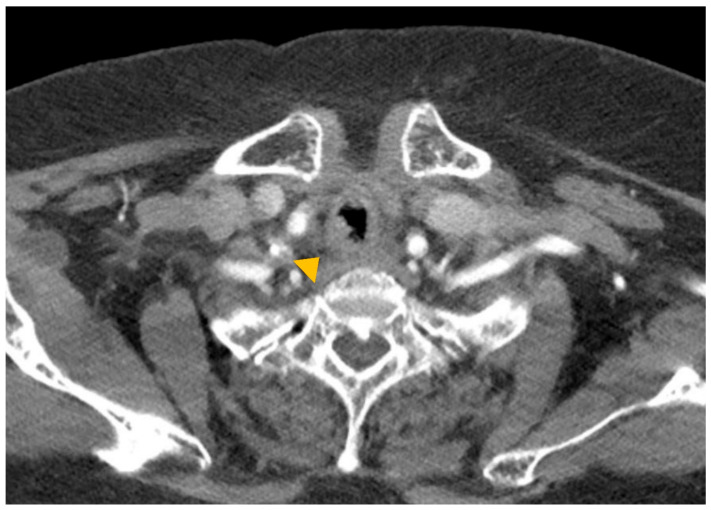
Neck computed tomography image. A diffuse wall thickening and intraluminal irregularity are noted in the proximal trachea (arrowhead).

**Figure 2:**
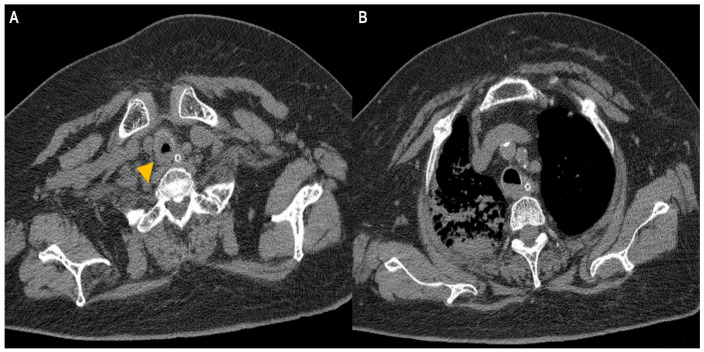
Chest computed tomography images. (**A**, arrowhead) Aggravated diffuse wall thickening with luminal narrowing at the proximal trachea. (**B**) Peribronchial consolidation and secretion stasis in the right upper lobe.

**Figure 3:**
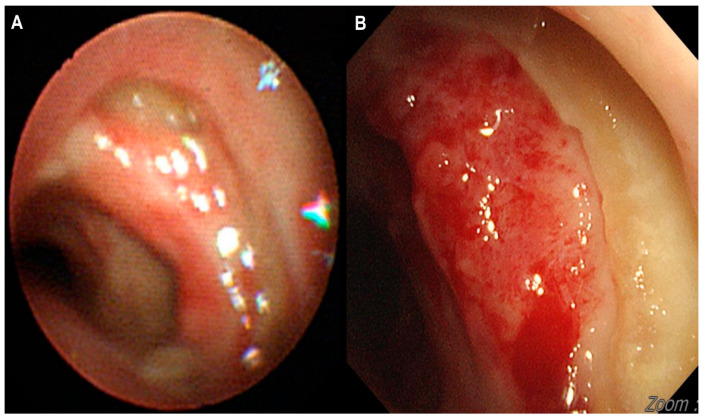
Bronchoscopic images. (**A**) Multiple deep ulcerations are noted in the proximal trachea. (**B**) Multiple biopsies were performed at the ulcer base and elevated ulcer margin.

**Figure 4:**
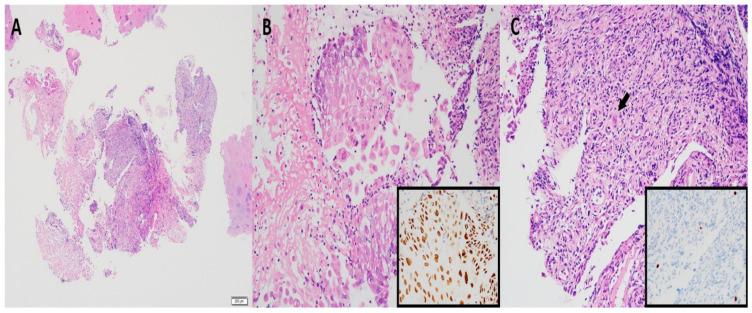
Representative images of hematoxylin and eosin and immunohistochemical staining (IHC). (**A**) An ulcer with granulation tissue and reactive squamous cell atypia is observed. (**B**) The keratinocytes present with the viral cytopathic changes of ground glass nuclei, nuclear molding, and multinucleated giant epithelial cells. IHC using the herpes simplex virus antibody was positive (inset). (**C**) An ulcer and granulation tissue with viral infected cells are noted (arrow), which were positive for cytomegalovirus (inset). The blue color indicates the hematoxylin counterstain. Original magnification, (**A**), ×40, (**B**,**C**), ×200 (Inset, IHC).

**Figure 5:**
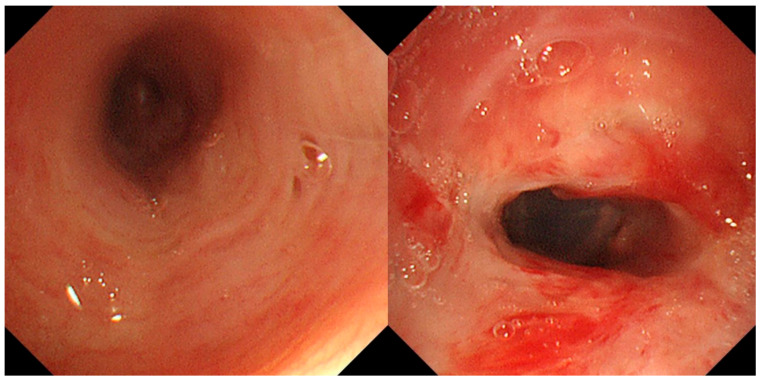
Follow-up bronchoscopic images. Improved multiple ulcerations with scars are noted in the proximal trachea.

A 74-year-old woman was admitted to our hospital with dyspnea and chest pain. She had hypertension, type 2 diabetes mellitus, heart failure, persistent atrial fibrillation, and chronic obstructive pulmonary disease (COPD). The patient did not use an inhalant for COPD because of low adherence to inhalation therapy. Human immunodeficiency virus testing was negative. On the second day of admission, she complained of chest pain and showed a decrease in consciousness. Torsade de pointes and ventricular fibrillation were observed. She underwent intensive care unit (ICU) treatment for 10 days, including mechanical ventilation, direct-current cardioversion, and a temporary pacemaker. The patient received prednisolone from 5 mg to 10 mg per day with inhaled budesonide/salbutamol/ipratropium for two months from the day of ICU admission to treat the COPD exacerbation and control dyspnea. After being transferred to the general ward, the patient remained stable with tapering and discontinuation of steroid administration. The patient developed throat discomfort on the 68th day of hospitalization. Since then, her dyspnea gradually worsened without desaturation and CO_2_ retention. On the 76th day of hospitalization, the patient complained of sustained throat discomfort and dyspnea with stridor. Neck computed tomography revealed a narrowing of the proximal trachea with a diffuse wall thickening and intraluminal irregularity (Figure 1), and video laryngoscopy detected a tracheal mass-like lesion. On the same date, acute respiratory failure occurred (the oxygen saturation by pulse oximeter was 56%, and the respiratory rate was 32 breaths per min). CO_2_ retention was detected in the arterial blood gas analysis (pH: 7.037, PaCO_2_: 66.1 mmHg, PaO_2_: 205.3 mmHg) at 15 L/min via reservoir bag. The patient was transferred to the ICU for mechanical ventilation. There was no evidence of nosocomial pneumonia in the chest X-ray, and serum C-reactive protein was normal (<0.5 mg/dL). No severe obstructive respiratory pattern was observed on the flow graph of the ventilator monitoring, and the CO_2_ retention immediately improved after endotracheal intubation in low grade pressure support. On the second day after intubation, chest computed tomography showed an aggravation of the multifocal diffuse tracheal wall thickening with luminal narrowing (Figure 2A) and a new-onset peribronchial ill-defined consolidation in the right upper lobe (Figure 2B). The piperacillin-tazobactam was initially administered for treatment of pneumonia. Bronchoscopy revealed a proximal tracheal narrowing with multiple ulcerations of the tracheal mucosa surrounded by an erythematous margin (Figure 3A). Multiple biopsies were performed at the base and margin of the ulceration (Figure 3B). Microscopically, reactive squamous atypia was observed in the background of the ulcer and granulation tissue (Figure 4A). In the squamous epithelium, numerous keratinocytes with virus-infected changes, including ground glass nuclei and nuclear molding, and multinucleated giant cells were observed (Figure 4B). Immunohistochemistry showed positive results for the anti-HSV antibody. A few viral cytopathic cells were noted in the granulation tissue, which were positive for the anti-CMV antibody (Figure 4C). The CMV real-time polymerase chain reaction (PCR) in the bronchial alveolar lavage fluid was also positive (31,775 copies/mL). Therefore, intravenous ganciclovir (300 mg twice daily), which is effective against HSV and CMV, was administered for 44 days. Despite the cessation of the sedative drugs, the patient’s consciousness did not improve. Brain magnetic resonance imaging revealed a multifocal petechial hemorrhage in the bilateral temporo-parietal lobe and left insula and minimal subdural hygroma along the left cerebral convexity. These findings were consistent with hemorrhagic encephalitis. The cerebrospinal fluid examination was within normal limits (white blood cells, 1 cells/µL; protein, 43 mg/dL). The viral PCR results for enterovirus, CMV, HSV, and varicella-zoster virus were all negative. On the 19th day after intubation, a percutaneous dilatational tracheostomy was performed for the tracheal stricture. The patient was transferred to the general ward on the 29th day after ICU readmission. A follow-up bronchoscopy was performed about every two weeks. On the 44th day of ganciclovir therapy, the follow-up bronchoscopy showed an improvement in the ulceration with scars (Figure 5). Microscopically, no lesions were suspected from viral infection.

## 3. Discussion

Isolated viral tracheitis rarely occurs in adults and is often asymptomatic or presents with mild symptoms [1]. However, serious necrotizing tracheitis has been reported in adults [6,7]. On autopsy, necrotizing tracheitis of a focal or extensive nature due to 2009 H1N1 influenza A viral infections was observed in all decedents with a concurrent lower respiratory tract involvement, whose trachea was investigated [6]. Although rare, various viral agents causing tracheitis, including HSV, varicella-zoster virus, CMV, or measles virus, have been described in adults, which are linked to the individual predispositions of the host [2].

HSV causes tracheitis in adult patients who are immunocompromised, on mechanical ventilation, corticosteroid administration, and who have acquired immunodeficiency syndrome (AIDS) [8,9]. There are limited reports regarding isolated tracheitis caused by HSV. Alvarez–Uria et al. described that isolated tracheitis due to HSV, diagnosed by culture and PCR for type 1 HSV with the biopsied tracheal tissue, occurred in a young man who had been administered steroids due to bronchial asthma [10]. In particular, most other cases of HSV tracheitis were associated with mechanical ventilation [8,11,12]. Similar to our case, St. John et al. reported that isolated herpetic tracheitis with a significant narrowing of the distal trachea occurred in patients who had been receiving mechanical ventilation due to respiratory failure caused by COPD exacerbation [13]. Herpetic tracheitis caused weaning failure, but the patient was successfully extubated following antiviral therapy [13]. The tracheal mucosa which is locally damaged by mechanical ventilation may be vulnerable to viral infections. Tracheal mucosal erosions result in squamous metaplasia of the epithelium, as observed in our case, causing the virus to infect it more easily [11,14]. Tracheal intubation is also likely to induce HSV spreading due to the aspiration of oral secretions, which contain the HSV due to the preceding HSV stomatitis. In addition, prolonged mechanical ventilation facilitates herpes reactivation as a result of temporary immunodeficiency or concurrent corticosteroid therapy. In our case, the patient was suspected to have concurrent viral encephalitis on brain imaging. However, it was difficult to determine its cause because the cerebrospinal fluid was negative for HSV and CMV on PCR.

Isolated tracheitis due to CMV infection is extremely rare. There have been reports of bronchitis caused by CMV in patients who underwent lung transplantation, and of CMV pharyngitis in a patient with AIDS [15,16]. However, to the best of our knowledge, there has only been one case of isolated necrotizing tracheitis, and this was detected in a patient with AIDS [17]. There is much evidence of the potential for serious infectious complications, including CMV infections, in patients with COPD treated with corticosteroids [18]. In our case, the chronic use of corticosteroids and history of mechanical ventilation impacted the development of tracheitis caused by CMV and HSV. Synchronous infections of CMV and HSV can occur because these infections share predisposing factors. There were many cases of CMV and HSV coinfection in the skin, gastrointestinal tract, genital organs, and central nervous system [19,20,21,22]. Intranuclear and intracytoplasmic inclusions in the pathology of the tracheal mucosa are specific findings for CMV tracheitis [17]. CMV tracheitis was confirmed by PCR or a culture of CMV from biopsied tissue or tracheal washings.

## 4. Conclusions

We present a case of infectious tracheitis caused by coinfection with CMV and HSV in a chronic steroid user due to COPD. CMV and HSV tracheitis can cause respiratory failure. CMV and HSV tracheitis was diagnosed based on the inclusion body and typical cytopathologic lesions, respectively, and the immunohistochemistry was positive for a tracheal ulcer pathology. Notably, isolated CMV tracheitis in patients without AIDS has not been previously reported. This case suggests that CMV and HSV may cause serious tracheitis, leading to respiratory failure.
